# Impact of *d*-states on transition metal impurity diffusion in TiN

**DOI:** 10.1038/s41598-023-34768-7

**Published:** 2023-05-22

**Authors:** Ganesh Kumar Nayak, David Holec, Martin Zelený

**Affiliations:** 1 Department of Materials Science, Montanuniversität Leoben, Franz-Josef-Strasse 18, 8700 Leoben, Austria; 2 Faculty of Mechanical Engineering, Institute of Materials Science and Engineering, Brno University of Technology, Technická 2896/2, 616 69 Brno, Czech Republic; 3 Present Address: Materials Chemistry, RWTH Aachen University, Kopernikusstraße 10, 52074 Aachen, Germany

**Keywords:** Ceramics, Metals and alloys, Atomistic models, Computational methods

## Abstract

In this work, we studied the energetics of diffusion-related quantities of transition-metal impurities in TiN, a prototype ceramic protective coating. We use ab-initio calculations to construct a database of impurity formation energies, vacancy-impurity binding energies, migration, and activation energies of 3*d* and selected 4*d* and 5*d* elements for the vacancy-mediated diffusion process. The obtained trends suggest that the trends in migration and activation energies are not fully anti-correlated with the size of the migration atom. We argue that this is caused by a strong impact of chemistry in terms of binding. We quantified this effect for selected cases using the density of electronic states, Crystal Orbital Hamiltonian Population analysis, and charge density analysis. Our results show that the bonding of impurities in the initial state of a diffusion jump (equilibrium lattice position), as well as the charge directionality at the transition state (energy maximum along the diffusion jump pathway), significantly impact the activation energies.

## Introduction

Diffusion plays an important role in the properties of solids which governs the kinetics of microstructural changes and processes of mass transport. Titanium nitride (TiN) and its alloys with various impurities have proved promising in the market^1–4^. Several diffusional processes can arise in these alloys and thus influence their mechanical performance, mainly at elevated temperatures. Especially diffusional phenomena are most widespread in metallic and ceramic systems. For example, creep deformation occurs at a rate dependent upon diffusional rearrangement of atoms at dislocation cores, e.g., changes in the composition of the inter-granular phase due to oxidation and grain-boundary diffusion^5–7^. Especially in TiN, the diffusional creep is suggested as a creep-controlling mechanism, and in this diffusion within the grains allow the ceramic to yield an applied stress^5^. Oxidation^8^ and intermixing^9^ with protective coatings are other examples of diffusional phenomena relevant to the operation of these compounds.

TiN is a widely used ceramic material that crystallizes in the face-centered cubic (fcc) B1 structure. Composition-wise, it has nitrogen content typically between 37 and 50 at.%^10^. Its high strength-to-weight ratio, high hardness (>25 GPa^11,12^), high melting point ($\approx$ 3220 K^13^), good wear and corrosion-resistant properties^14^ and excellent chemical inertness against many workpiece materials make it a perfect prototype protective hard coatings candidate to protect underlying surfaces on cutting tools. However, with time and emerging materials development, the scientific effort has been accomplished in TiN-based ternaries. With the basic building block of TiN ceramic, the additional and suitable transition metals with adjusting ratios offer various possibilities to tune the different properties, especially structural, mechanical, electrical, lubricant, etc. The growth and processing parameters are optimized to control stoichiometry, microstructure, and texture. For example, alloying element Mo, W^1^ in TiN are reported in the supertoughening process in ordered B1. And also, the impurity alloy Ta and Nb^2^ in TiN has been reported recently. Similarly, Yttrium has shown enhanced oxidation resistance, and hardness^15^, alloying of Zr has significantly improved the adhesive strength of TiN-based coatings to the substrate^16^, Cr and Hf have emerged as alloying elements for the higher wear resistance, and hardness^17–19^, alloying of V is a possible candidate for self-lubricant coatings^4^,

Here we are more interested in the diffusion process of the above-discussed alloying element in different circumstances. The circumstances may mimic the spinodal decomposition, high *T* diffusion such as in self-lubricant coatings, or diffusion in the high-entropy (HE) based alloys, etc., where the diffusion is crucial. Particularly, we are interested in quantifying the diffusion barriers of single transition metal impurity (TMI) in the B1 TiN single crystal model. Two important factors motivate us to conduct this study: (i) a database of diffusion-related quantities of TMIs in TiN and (ii) *d*-states effect of TMIs on their diffusion in TiN.

There is a gap in the literature for a diffusion-related database of various TMIs in the host TiN alloy. The various quantities are impurity formation energies, solubilities, vacancy-impurity binding energies, diffusion migration barriers of impurity, and activation energy. Understanding various physical and chemical factors influencing these quantities is vital for developing and designing new TiN-based ternaries. Such data is available for many technologically important host alloys such as Al, Mg, Cu, Co, and Ni^20–31^. Nonetheless, all these diffusion-related data fill the database as a single metal host lattice, even though most are fcc structures. A similar diffusion database is unavailable extensively for B1 binaries, especially for any nitrides; it includes experiments and theory. Because the B1-TiN structure contains one sub-lattice of Ti and another one of N, the diffusion behavior of TMIs could possibly be different than single metal host lattice. There are studies on diffusion on Ti in TiN^1,3,4^, Ti in AlN^9^, V in TiN^4^, V in VAlN^32^, Ni in TiN^33^, Cu in TiN^34–36^, but this is not the whole picture of TMIs diffusion database in energies.

Another perspective of this work is the *d*-states effect on the diffusion of TMIs. In our previous work, we studied the V-impurity and Ti-self diffusion in the same B1-TiN single crystal model^4^; we quantified not only the 0 K migration barriers but also the activation energy and pre-exponential factor using finite temperature calculations by quasi-harmonic approximation. The contradiction arises in our study in diffusion value of V and Ti; a smaller atom yields higher migration or activation energy and hence a lower diffusion coefficient. A common expectation is that the smaller atom has a larger diffusion rate and lower activation energy. On the other hand, Janotti et al.^21^ claims that the trend in diffusion is such that the larger atom can move faster irrespective of metallic host lattices. And they justified this phenomenon as compressibility of *d*-states of metallic bonding directionality. But in our case, we are more concerned about the ceramic host and/or the host having a mixed bonding character (ionic, metallic, and covalent). And hence we start moving toward interpreting the *d*-orbitals effect of the considered *d*-impurity on the diffusion in TiN (a ceramic host). In this type of host, apart from compressibility, we presume the bondings for different *d*-orbitals and charge transfer have a major role in such a phenomenon.

The present work aims to quantify and create a database for the diffusion energy migration barriers ($E_m$) of different group transition impurities in B1 TiN, along with the energy affecting or related to the diffusion. This study gives insight into the diffusion phenomena in coating systems with *d*-impurity alloying elements. On the contrary, this study perceives the effect of *d*-orbitals correlation on the diffusion-related energies while coming to the migration of *d*-impurities. This can lead us to a conclusion about whether the misfit lattice–strain plays the key role in the rise of diffusion migration barriers in TiN or whether there is a significant role of *d*-orbitals in such materials.

We employed 0 K Density Functional Theory (DFT) calculations to estimate the energies and perform bond analysis for bulk diffusion of all 3*d*, a few 4*d*, and 5*d* impurities in TiN. We discuss the energetics of the single *d*-impurity in TiN and relate them to the atom size in section Energies. Section Bonding characteristics contains the representative analysis of the impact of *d*-states on the electronic structure and bonding.

## Results and discussion

### Energies

The B1-TiN structure has two sublattices, one belonging to Ti and another to N. We focus on Ti substitutions on the metal sublattice. The impurity atoms under consideration in this article include the complete 3*d* row (including magnetic Co, Cr, Fe, Mn, Ni, and all other non-magnetic species), and selected examples of 4*d* and 5*d* substituents.

The calculated energy values are listed in Table 1. The single impurity formation energy, $E_{imp}^f$ (Eq. 1), signifies the amount of energy needed to supply to remove the impurity atom from the TiN-host matrix. The positive energy corresponds to the unfavorable nature of such substitution (w.r.t. the chosen reference states). $E_{imp}^f$ of *d*-impurity follows the increasing trend with the atomic number in each row. For example, among the 3*d*-impurities, the Sc-atom shows a favorable nature in TiN (negative $E_{imp}^f$), and starting from Ti to Cu, the energy gradually increases to positive values. Interestingly, Cu to Zn does not fit this trend, i.e., Zn is a more stable substitution than Cu, possibly because of the filling of having a fully filled *d*-orbital shell. Among the studied 4*d* and 5*d* impurities, only Zr and W, respectively, seem to be outliers from the trend. We will discuss origins for such out-of-the-trend behavior by performing a bond analysis in section Bonding characteristics.

**Figure 1:**
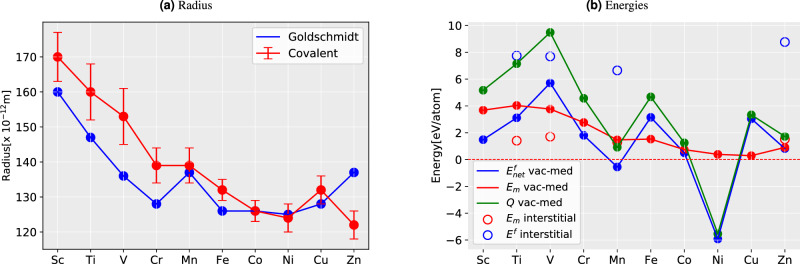
Comparison of the size of the atom and the energy in 3*d*-impurities (**a**) the Goldschmidt radii (i.e., atomic radii)^38^ and covalent radii^39^ of the corresponding impurity in TiN (**b**) Migration barriers ($E_m$), formation energies ($E^f$, $E_{net}^f$), and the activation energy (*Q*) for the vacancy-mediated process of all the impurities and that of interstitials of selected impurities.

**Figure 2:**
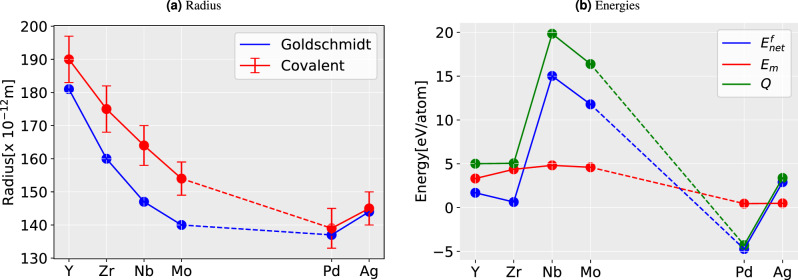
Comparison of the size of an atom and the energy in 4*d*-impurities. (**a**) The Goldschmidt radii (i.e., atomic radii)^38^ and covalent radii^39^ of the corresponding impurity in TiN (**b**) Migration barriers ($E_m$), net formation energy ($E_{net}^f$), and activation energy (*Q*).

**Figure 3:**
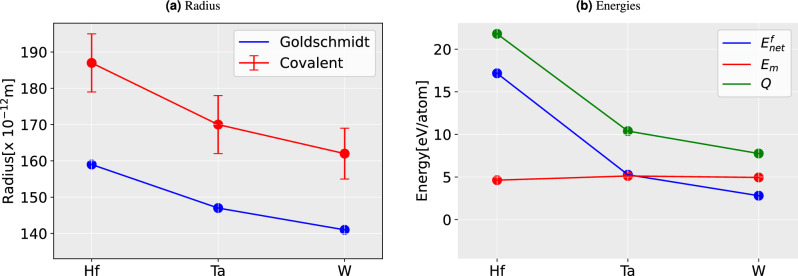
Comparison of the size of atom and the energy in 5*d*-impurities. (**a**) The Goldschmidt radii (i.e., atomic radii)^38^ and covalent radii^39^ of the corresponding impurity in TiN (**b**) Migration barriers ($E_m$), net formation energy ($E_{net}^f$), and activation energy (*Q*).

In our work^4^, we showed that V and Ti diffusion in TiN is dominated by the VM mechanism over interstitial one, which was also studied earlier by Glicksman^37^. Besides, in the VM mechanism, bonding analysis of both the initial state (IS) and transition state (TS) can be performed. For instance, the impurity atom perfectly sits in the lattice site in IS and provides insight into the bonding character. On the other hand, in the TS, the correlation of *d*-states also can be analyzed. The vacancy formation energy, $E^f$, of a single Ti-vacancy, is 3.11 eV/atom in TiN (Eq. 3). Since we are about to form a vacancy–impurity pair for the VM migration mechanism as elaborated in section Energy estimation in the Methods section, additional energy $E_{bind}$ will contribute to the formation of energy calculated through the Eq. (5). We then sum up the $E^f$ and $E_{bind}$ to get $E_{net}^f$. All of the values are listed in Table 1. Additionally, $E_{net}^f$ is plotted in Figs. 1b,  2b and  3b for impurity 3*d*, 4*d*, and 5*d*, respectively. There is no particular trend observed for the $E_{net}^f$ for any *d*-impurity series. However, $E_{net}^f$ is the $E_{bind}$ shifted by the Ti-vacancy formation energy, $E^f$. This signifies that the different impurities bound with the vacancy very differently. We note that such binding modification will impact both the diffusion and mechanical properties of a solid. The contribution mainly comes from the misfit of the atom in terms of *d*-states bonding. In this formulation, negative $E_{bind}$ correspond to attractive interactions of the vacancy–impurity, whereas positive $E_{bind}$ correspond to the repulsive interactions. For example, the highest binding energy in the 3*d*-series is for V, while the lowest is predicted for Ni. Interestingly, Ni has the most negative $E_{bind}$ of all investigated impurities here, and hence, vacancy acts as the strongest binder in this case.

**Table 1:** The calculated values of all the energies related to diffusion and forming of an impurity atom in B1-TiN for all 3*d* and selected 4*d* and 5*d* impurities.

Impurity	$E_{imp}^f \,\mathrm {(eV)}$	$E_{bind} \,\mathrm {(eV)}$	$E_{net}^f \,\mathrm {(eV)}$	$E_m \,\mathrm {(eV)}$	$Q\ \,\mathrm {(eV)}$
3*d*					
Sc	− 0.27	− 1.63	1.48	3.68	5.17
Ti	0.00	0.00	3.11	4.03	7.14
V	1.50	2.60	5.72	3.77	9.48
Cr	2.86	− 1.31	1.81	2.76	4.57
Mn	3.74	− 3.67	− 0.55	1.47	0.92
Fe	3.92	0.03	3.15	1.53	4.68
Co	4.03	− 2.61	0.50	0.74	1.25
Ni	4.60	− 9.04	− 5.92	0.39	− 5.53
Cu	4.63	− 0.07	3.04	0.29	3.34
Zn	4.14	− 2.29	0.82	0.90	1.73
4*d*					
Y	1.40	− 1.42	1.69	3.32	5.00
Zr	0.36	− 2.48	0.64	4.37	5.00
Nb	1.33	11.90	15.02	4.82	19.84
Mo	2.88	8.66	11.78	4.60	16.38
Pd	6.28	− 7.84	− 4.73	0.45	− 4.28
Ag	6.93	− 0.21	2.90	0.48	3.39
5*d*					
Hf	0.07	14.05	17.17	4.63	21.80
Ta	1.41	2.15	5.27	5.12	10.39
W	− 3.19	− 0.31	2.81	4.95	7.75

Note that the impurity formation energy, $E^f_{imp}$, is in eV/(impurity atom), $E_{bind}$ and $E_{net}^f$ in eV/(vacancy-impurity pair). And the $E^f_{net}$ for all the species is $E_{bind}$, scaled by the formation energy of single Ti vacancy($E^f$) as per the Eq. (6). The value calculated for $E^f$ is 3.11 eV/atom.

To complete our database for discussing the diffusion, we estimated the diffusion migration barriers, $E_m$, through minimum energy path (MEP) and activation energy, *Q*, with the help of Eq. (7). The values are also listed in Table 1. The $E_m$ is calculated by displacing an impurity atom towards the adjacent vacancy along the $\left<110\right>$ direction in the fcc Ti sublattice of B1-TiN.

The values of $E_m$ are plotted in Figs. 1, 2 and 3 along with the Goldschmidt radii (atomic radii)^38^ and covalent radii (with errorbar)^39^ for 3*d*, 4*d*, and 5*d* respectively. While estimating the $E_m$, one should deal with the energy difference between the impurity atom at the lattice site or IS and at the saddle point or TS. The bigger atomic radius refers to a bigger atom and hence a larger misfit or amount of contraction/expansion in the host lattice (w.r.t. host Ti atom), producing a larger strain and thereby contributing to (strain) energy increase in both IS and TS. One acknowledges the difference in strain will be evident in the change in inter-atomic distances.

The results suggest a qualitative correlation between the impurity sizes and their diffusion energy barriers, $E_m$ (see Figs. 1b,  2b,  3b). This is particularly true for the Goldsmitdt radius. Nevertheless, the agreement is also decent between $E_m$ and the covalent radii when considering the low-spin configurations, reflecting that TiN bonds exhibit a partially covalent character^40^. The results are thus rather straightforward in the 3*d*-series, i.e., the bigger the atom larger the energy barrier; a clear outlier from this trend seems to be Mn and possibly also Sc.

However, 4*d* and 5*d*-series do not show any clear correlation between the impurity size and the energies, $E_m$ or *Q*. In particular, the moment binding energy enters the picture, all the trends for all impurities drastically change. Since the *Q* is the sum of $E_m$ and $E_{net}^f$, with the former exhibiting a rather smooth trend, *Q* takes the shape of $E_{net}^f$ in most cases. This means primarily that the energy $E_{bind}$ impacts *Q*. To elaborate on the trends in $E_{bind}$, which is strongly related to the bonding, an analysis of the electronic structure will be needed (see section Bonding characteristics). Nevertheless, here we emphasize the significance of negative values of *Q* (see Table 1), formed of two contributions. The negative value of $E_{net}^f$ is directly related to the stability of different *d* impurity–vacancy pairs. The negative binding energies correspond to attractive interactions between the vacancy and the impurity, whereas positive binding energies correspond to repulsive interactions. Hence pairs with negative values are easier to diffuse via vacancy-mediated mechanism as compared to those with positive $E_{net}^f$.

Regarding the barriers $E_m$, however, we cannot ignore some degree of their correlation with atomic size. For instance, in 4*d*, and 5*d* (see Figs. 2, 3) suggest that larger impurity radius results in lower $E_m$. This trend is less prominent in 3*d* metals (see Fig. 1). However, when it comes to the pairs Sc–Ti, Cr–Mn, and Mn–Fe, the trend can be seen clearly again(see Fig. 1b). These pairs have the same trend of *Q* as $E_m$, but in the case of 4*d* and 5*d*, the trends for *Q* change. We note that the major contribution to the variation of the *Q* across the periodic table rows comes from the variation of the $E_{bind}$ and is inconsistent for different *d*-series of impurity. The results also reveal that an increasing atom size can lead to a decrease in $E_m$ and *Q* in TiN, but not always. On the other hand, *Q* for Ni and Pd has a negative value, which is contributed from their $E^f_{net}$. This indicates the instability of the system with these alloying elements in it. The decomposition of such system has previously been studied both for Ni^41–43^ and Pd^44,45^ with rich N environment, and nitride of both (Ni, Pd) has been recognized as a metastable compound.

### Bonding characteristic affecting energies

We propose that the origin of the diffusion trends lies in the characteristics of the occupied *d*-states of impurities. Therefore, to shed some light on the puzzling relations between atom sizes and the migration energetics, we examine the bonding characteristics of the impurity atoms in the host lattice. In the case of the TMI series in the particular group, the smaller radius is coupled with poor shielding capacity, especially for the midrow elements^46^. 4*d* and 5*d* impurities have a larger radius than the 3*d* impurities due to better shielding. As a result, the nuclear charge strongly influences the directional bonding of 3*d* atoms. Hence, to amplify the effects, 3*d* impurities are chosen for the critical assessment of directional bonding. Namely, we exemplify our analysis on Sc–Ti and Mn–Fe pairs from the 3*d*-series impurities for which trends of *Q* (and to a smaller extent also of) $E_m$ anti-correlate with atomic radius. Additionally, these two pairs differ in the magnetic state of the interaction, i.e., non-magnetic Sc–Ti and magnetic Mn–Fe.

The activation energy *Q* has two major components: (i) the energy contributed from the directional bond or charge distribution (enters to *Q* in the form of $E^f_{net}$) and (ii) from the energy difference between IS and TS (enters to *Q* in the form of $E_m$). Hence the activation energy should reflect the bonding characteristics in the IS and the degree of bonding directionality of the impurity near TS.

#### Perfect TiN matrix

**Figure 4:**
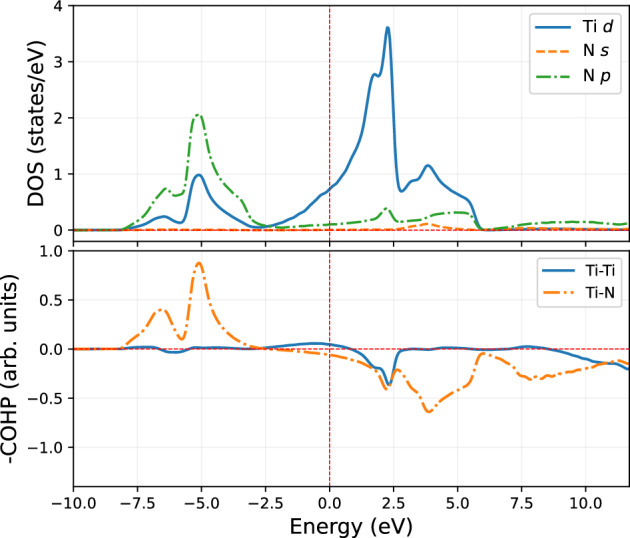
DOS (upper panel) and COHP (lower panel) analysis for bulk B1-TiN resolved in first nearest neighbor (1*NN*) Ti-N and second nearest neighbor (2*NN*) Ti-Ti orbital interactions. The zero energy level corresponds to the Fermi energy, $E_F$.

Let us first look at the difference between non-directional and directional bonds in the IS and their contributions to $E_{bind}$. Before introducing impurities into bulk TiN, we thoroughly analyzed its electronic structure, DOS and COHP. This will help us to identify the changes due to introducing vacancy and impurity together. The analysis shown in Fig. 4 confirms the mixed covalent Ti-N and metallic Ti-Ti bonding nature in TiN, in line with similar previous results by Yu et al.^40^. They claim the covalent bond density increases with the increase of N content (i.e., covalent interactions become stronger). In summary, the TiN host is metallic as Ti-Ti contributes to the metallic character; additionally, the overlap of the Ti-N orbitals increases (hence the covalent bond becomes stronger) in the presence of a single Ti-vacancy (as needed for the VM diffusion)^40^.

#### Bonding of 3*d* non-magnetic impurities

**Figure 5:**
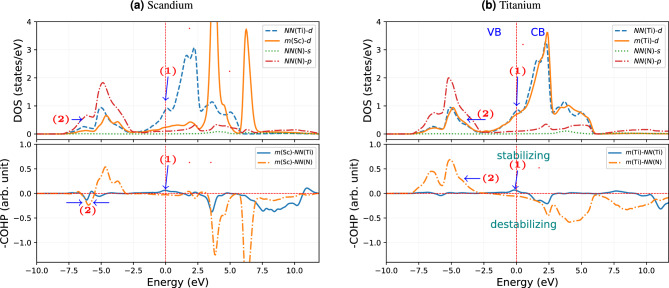
The interactions comparison of migrating (*m*) atom with surroundings 2*NN* Ti atoms, and 1*NN* N atoms for the IS of Sc and Ti impurity with a vacancy at the 2*NN* in TiN. The zero energy level corresponds to the Fermi energy.

**Figure 6:**
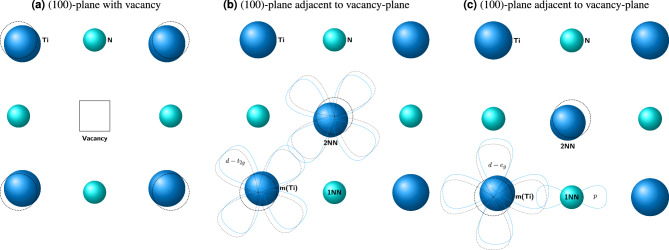
The schematic representation of the TiN system (**a**) with shifting of *NN*(Ti) atoms in the presence of a vacancy in (100)-plane, (**b**) change in the interaction of *m*(Ti) with surroundings 2*NN* Ti atoms in (100)-plane, adjacent to vacancy-plane, and (**c**) change in the interaction of *m*(Ti) with surroundings 1*NN* N-atoms in (100)-plane, adjacent to vacancy-plane.

Figure 5 shows the DOS and COHP, which manifest a qualitative comparison of the strength of bonding interactions of non-magnetic pair Sc and Ti in TiN with a vacancy adjacent to them. This argument helps us to understand the lower *Q* in the case of Sc than for Ti. In comparison to bulk TiN (see Fig. 4), the bonding interaction between the migrating Ti atom (*m*(Ti)) with its nearest neighbors (*NN*) (both Ti and N) in the TiN with vacancy has clearly changed (see Fig. 5b). For instance, an additional peak (labeled as (1) in Fig. 5b) appears near the $E_F$, strengthening the metallic bonding interaction between *m*(Ti)–*NN*(Ti) (in comparison to the Ti–Ti interaction in bulk TiN, Fig. 4). This is caused by an increased overlap of the *d*-states because *m*(Ti) is displaced towards the formed Ti-vacancy in its 2*NN* (see Fig. 6a). This can be realized from a distance between *m*(Ti) and *NN*(Ti) atom opposite to vacancy in 2*NN*, which decreases by $0.07$ Å w.r.t. pure TiN (see Fig. 6b). However, the covalent bonding of *m*(Ti)–*NN*(N) is weakening in comparison to bulk TiN, as one can be observed from a pronounced decrease of the peak height of the COHP curve in the energy range $-5.0$ to $-2.5$ eV and broadening to higher energy (labeled as (2) in Fig. 5b). The reason is again displacing *m*(Ti) towards the vacancy, as reflected in the shortening of Ti–N bonds near vacancy and elongating on the opposite side by $0.05$ Å. The change in overlap interaction of *m*(Ti) and *NN*(N) is demonstrated in the Fig. 6c.

In the case of *m*(Ti), there is a slight weakening of the covalent bond but a strengthening of the metallic bond w.r.t. bulk TiN. However, no evidence exists of forming (occupied) antibonding states compared to bulk TiN. Regarding Sc in TiN in the IS, there is a substantial destabilizing character in both covalent and metallic bonding. This is because of the pronounced shifting of *d*-states to the higher energy. This can be seen in the energy range between $-7.5$ and $-5.0$ eV of the COHP curve (labeled as (2) in Fig. 5a). These changes are also reflected in integrated COHP (ICOHP) values: $-2.98\, \,\textrm{eV}$ for Ti–N and $-2.65\, \,\textrm{eV}$ for Sc–N, pointing towards a stronger interaction with surrounding N in the case of the Ti atom. The ICOHP values for Ti–N in bulk TiN are $-2.98\, \,\textrm{eV}$ as well, which indicates the Sc–N covalent interaction is weaker. The above-discussed weaker bonding of *m*(Sc) compared to *m*(Ti) in the IS reveals the electronic origin of lower *Q* in the case of Sc migration compared to Ti.

**Figure 7:**
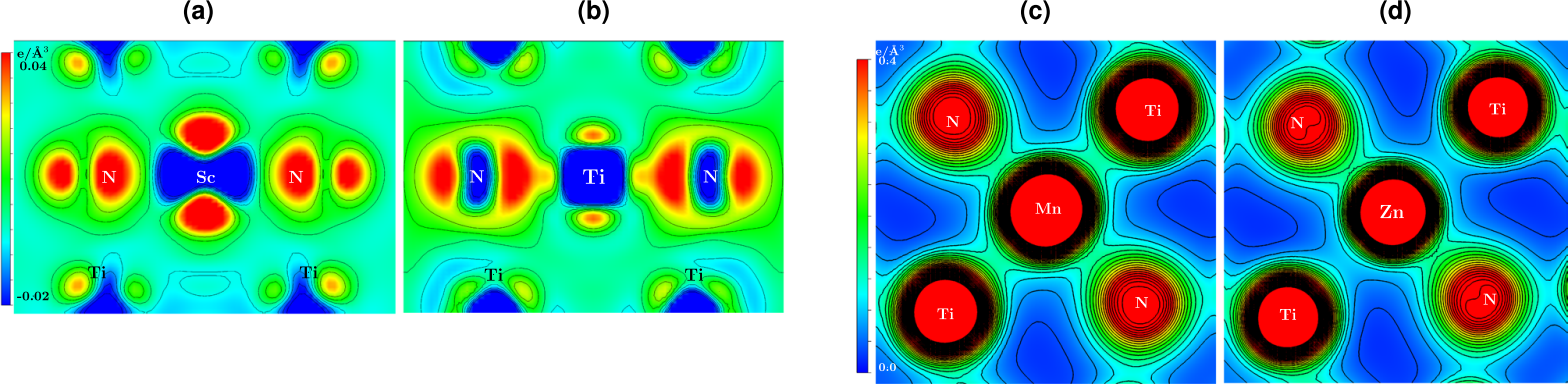
Charge density difference map of the different TS. The charge density difference map for the (**a**) Sc and (**b**) Ti in their TS (in the (101) plane) for VM mechanisms. The charge density plot for interstitial (**c**) Mn and (**d**) Zn in their TS, in the (100) plane. All plots are described in the unit of $\textrm{e}^{-}$/Å^3^.

Not only the IS but also bonding in the TS state significantly impacts the migration behavior. Figure 7a,b show the charge density difference maps (between superposition of atomic charges and charge density of real interacting atoms) for Sc and Ti in TS. In the case of Sc, the charge accumulates on surrounding N atoms, presumably suggesting an ionic character of the bonds. Electrons are also redistributed into the areas perpendicular to the Sc–N direction, hence not participating in the bonding. On the contrary, there is a large charge accumulation between Ti and N atoms (Fig. 7b), suggesting a covalent character of the Ti-N bond even in the TS. Consequently, the migration barrier of Ti is larger than that of Sc.

In contrast to the VM mechanism, the migrating atoms are more squeezed in the void of TS during interstitial diffusion and hence are presumably more correlated (form bonding) due to charge overlap. Hence, we have calculated the migration barriers for the interstitial mechanism for selected cases and show them with open circles in Fig. 1b. This data suggests that Zn has a higher $E_m$ than Mn despite having a smaller covalent radius (although their Goldsmitdt’s radius is the same). Moreover, Mn has an exact half-filled 3*d* shell while Zn has a fully-filled 3*d* orbitals. Figure 7c,d represent the electron distribution of Mn and Zn in their respective TS of the interstitial mechanism. The presence of (smaller) Zn-atom pushes the *NN*-Ti to a farther distance compared to Mn. Measured by the distances of surrounding Ti-N bonds, their lengths are 2.68 and $2.8$ Å for Mn and Zn, respectively. We propose that this repulsion is a demonstration of the Pauli repulsion due to the fully-filled *d*-orbitals. Clearly, there is minimal overlap (and hence bonding) between Zn and Ti, unlike in the case of Mn and Ti.

#### Impact of magnetism

**Figure 8:**
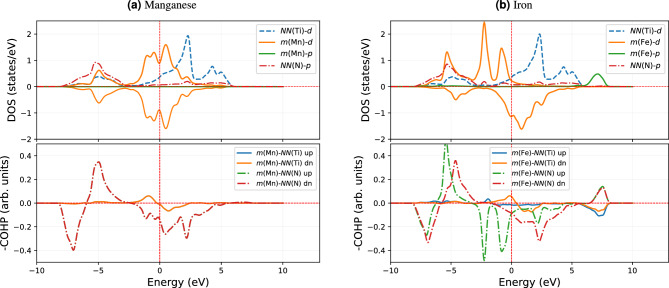
The interactions comparison of migrating atom with surroundings 2*NN* Ti atoms, and 1*NN* N-atoms in terms of DOS (upper panel) and COHP (lower panel) for the IS of Mn and Fe impurity with a vacancy at the 2*NN* in TiN. The lower and upper regions of DOS are divided into minority-spin (dn) and majority-spin (up).

Let us verify the correlations between migration energetics and bonding drawn in the previous section based on the electronic structure analysis of the IS also for magnetic systems. We choose the 3*d* (magnetic) impurities Mn and Fe, neighbors in the periodic table. For a magnetic system, the occupancy of majority and minority spin states plays a role in the bonding separately. Figure 8 presents DOS and COHP of majority (up) and minority (dn) spins. Foremost we note that the Mn COHP and its DOS show no magnetic splitting, and hence no separate contributions come from the minority and majority spin components.

This is not the case for Fe: there is a shifting of minority spin states towards $E_F$ and partly to the conduction band. This means some of the metallic state electrons start filling the valence states. This stabilizes the covalent and metallic interaction, which also appears in the bonding states. This is absent in the case of Mn. The *d* states of majority spin shift towards lower energies for Fe in comparison to Mn. As a result, Fe interaction with *NN*(Ti) is stabilized. This is evident by the bonding states close to $\approx -2.5\,\textrm{eV}$ shown in the COHP plot. On the other hand, the minority spin states shift towards the $E_F$, and only a small fraction of these states are actually occupied (i.e., below $E_F$). Due to the minority state shifting towards the higher energy, the antibonding covalent states formed in Fe with *NN*(N). However, the minority spin states also stabilized the bonding metallic states near $E_F$. This is accompanied by a stabilizing interaction between *m*(Fe)-*NN*(Ti), as revealed by COHP as a small peak near to $E_F$. Nevertheless, due to magnetic state splitting, the covalent bonding states in Fe splits into two in the COHP. The green curve in the lower panel in Fig. 8b comes from the majority spin states, whereas the red one corresponds to the minority spin. Comparing Mn and Fe, we, therefore, conclude that the magnetic splitting of Fe leads to a stabilization of its IS state and hence contributes to the *Q*.

#### Final note

From the above-presented electronic structure analysis of both, magnetic and non-magnetic cases, it is evident that the migration energetics are significantly influenced by the *d*-states correlation of the migrating species with the matrix atoms. This applies not only to the IS, but also to the TS, as demonstrated with the example of bonding directionality in the VM-(Sc–Ti) and interstitial-(Mn–Zn) migration case, we also showed the contribution from TS has a finite contribution from the directionality of *d*-states and its correlation. This evidence is clear from the difference in interatomic distances of *NN*(N). Both in IS and TS of Sc and Ti in TiN is 0.07 Å, which is a very weak dependence between the two structures. Janotti et al.^21^ elaborated this as the larger *d*-impurity atoms have more compressibility and give rise to lower barriers. However, the host material in their study was a metallic system. Unlike that, we discussed an example of ceramic TiN, where ionic, covalent, and metallic bonds are in the play, and hence an orbital compressibility argument alone does not suffice.

## Conclusions

In this study, we have created a database of energy contributions to the activation energy of transition metal (*d*-states) impurity diffusion in TiN. The obtained results were rationalized in terms of the density of electronic states and crystal orbital hamiltonian population (COHP). The main conclusions drawn from this work are summarised as follows:

- Smaller atom does not necessarily move faster (does not have small activation energy), and vice-versa. The faster or slower migration of *d*-states metal atoms sensitively depends on the bonding and anti-bonding interactions with the environment. In the TiN case, they are affected by host N and Ti atoms.
- Migration barriers are affected by both the initial and the transition states of the migration. The IS contributes by the different bonding arrangements of impurity’s *d*-states. In the TS, the energy is affected by the *d*-states directionality and correlation with the orbitals of the host atoms.
- The impurity-vacancy binding energy, and hence the activation energy, strongly depends on the bonding of the TMIs in the matrix.

This work provides a knowledgebase for understanding and designing diffusion-related behavior of *d*-impurities in TiN, such as spinodal decomposition, phase formation, etc. It also provides an insight into the interactions among the *d*-states in alloyed ceramic TiN.

## Methodology

### Energy estimation

The study is purely related to the *d*-impurity diffusion in the TiN. One needs to know how much energy they cost while alloying with TiN. Hence we attempt to calculate the impurity formation energy in a dilute limit, i.e., a single impurity in bulk TiN. This is the energy spent/released when an impurity is placed in a bulk host lattice, which will help us better understand TMIs’ behavior as an alloying element. Hence we plot the formation energy of an impurity in the host lattice, and we define this as,

1 $$\begin{aligned} E_{imp}^f = E_{imp} - E_{bulk} + \mu _{vac}- \mu _{imp} \end{aligned}$$

where $E_{imp}$ and $E_{bulk}$ are the energies of bulk with a single impurity replacing a host atom (here Ti) and bulk defect-free supercells, respectively, and $\mu _{vac}$ is the chemical potential of vacant (removed) species where additionally $\mu _{imp}$ is the chemical potential of the impurity atom placed in the place of vacant species. The $\mu _{vac}$ and $\mu _{imp}$ (the energies per atom of the metal) are calculated in thier respective ground-state structure 0 K(e.g., hcp-Ti, bcc-V, etc.).

Point defects and their energy in solids play a central role in materials physics and are key to understanding diffusion in solids. In a VM diffusion process, one adjacent site should be vacant to accommodate the migrating atom. Hence it’s crucial to know the probability of the vacancy site. The probability of finding an adjacent vacancy to an impurity,

2 $$\begin{aligned} p = C_0 e^{\left( \frac{E^f}{k_BT}\right) } \end{aligned}$$

where $C_0$ is the lattice coordination, $k_B$ is the Boltzmann constant, and $E^f$ is the formation energy of a single host atom. The Gibbs free energy of formation ($G^f$) is a characteristic of vacancies that governs the equilibrium concentration at any given temperature, which later enters the diffusion coefficient (*D*). But here, our calculation is restricted to 0 K, and we are not looking to estimate *D*. Since, in our case, we are only interested in the diffusion in the Ti sublattice, $E^f$ will be the vacancy formation energy of a single Ti-vacancy. This is given as,

3 $$\begin{aligned} E^f = E_{vac} - E_{bulk} + \mu _{vac} \end{aligned}$$

where $E_{vac}$ is the energy of a supercell with vacancy. The formation energy of interstitial impurities in TiN is calculated as:

4 $$\begin{aligned} E^f = E_{inter} - E_{bulk} - \mu _{inter} \end{aligned}$$

where $E_{inter}$ is the energy of the supercell with one additional interstitial atom.

However, when the impurity is accommodated adjacent to the vacancy, the interaction of the impurity and vacancy gives rise to additional energy. This energy is known as the vacancy–impurity binding energy and is defined as the following^47,48^:

5 $$\begin{aligned} E_{bind} = E_{vac,imp} - E_{imp} + E_{bulk} - E_{vac} \end{aligned}$$

In this equation, $E_{vac,imp}$ denotes the total energy of the supercell containing the impurity atom with a vacancy in its nearest-neighbor site. Here the energy $E_{bind}$ is determined as the difference between the vacancy formation energy in the vicinity of the impurity atom and the pure bulk, which can be seen in the formula.

From the above equations and analysis, it is clear that when the impurity diffusion is taken into account, the energy associated with it impacts the process. For example, $E^f$ determines the probability of vacant space, i.e., vacancy concentration. And from the definition, $E_{bind}$ is the additional vacancy formation energy when an impurity atom is present with a vacancy in the neighborhood. So the migration or jump to happen, there should be at least one adjacent vacancy available. Hence, both energy will add up to the net formation energy and will be given as,

6 $$\begin{aligned} E_{net}^f = E^f + E_{bind} \end{aligned}$$

When calculating the diffusion coefficient for vacancy-mediated impurity diffusion, this net formation energy enters the exponent in the Arrhenius equation as a contribution to the activation energy^4^ along with the Eq. (2).

Since we did not consider temperature-dependent contributions to free energy in our calculations, the net formation energy, $E_{net}^f$, along with migration energy, $E_m$, should contribute to the activation energy (*Q*),

7 $$\begin{aligned} Q = E_{net}^f + E_m \end{aligned}$$

The $E_m$ is the migration energy barrier achieved from the energy change of the system between IS and TS along the diffusion minimum energy path (MEP) calculated from 0 K DFT.

### Computational methods

The DFT calculations reported herein were performed with the Vienna Ab initio Simulation Package (VASP)^49,50^ in the generalized gradient approximation of GGA-PBE^51^, and the projector described the electron-ion interactions augmented wave potentials (PAW)^52^. Structure relaxations and MEP calculaions were carried out with 10 $\times$ 10 $\times$ 10 *k*-point grids^53^, along with the density of states (DOS), charge density distribution, and all other bonding-related calculations. The Methfessel–Paxton^54^ smearing of $0.2\,\textrm{eV}$ was applied in the process. In all calculations, we used a large energy cut–off of 500 eV for the plane wave basis to achieve total energy convergence within $10^{-6}\,\textrm{eV}$ (per supercell) during the electronic self-consistency electronic cycles. And for the ionic relaxations during structural optimizations, the total energy convergence of $10^{-4}\,\textrm{eV}$ (per supercell) was applied.

The TS corresponding to the saddle point along the diffusion MEP was determined using the nudged elastic band (NEB)^55^ method implemented in VASP. The diffusion pathway of MEP for the interstitial mechanism is along $\left<100\right>$ direction, and that of the VM mechanism is $\left<110\right>$ direction. We tested five images versus three images versus single image NEB calculations for the migration barriers and found the resulting barriers do not vary up to 4 decimal points. The size of the supercell in calculating the NEB was tested at 64 vs. 216 atomic cells, and the energy difference we found is $10^{-2}\,\textrm{eV}$. The formation energy difference in our test case was 0.03 eV for comparing 64 vs. 214 atomic cells. Hence even for the impurity/vacancy formation energy calculation, 64 atomic supercells proved sufficiently accurate for the present purpose. Hence, all the calculations are done in a cell containing 64 atomic sites and representing $2 \times 2 \times 2$ supercell of a conventional cubic B1 structure of TiN cell. We calculated the charge density of crystal structures in real space using 256 $\times$ 256 $\times$ 256 grid points. This technique of charge density calculations entails mapping the difference between charge density derived from a non-self-consistent calculation of a superposition of atomic charge densities and self-consistent charge densities derived for the whole system. This is thus useful in identifying the impurity atom’s charge distribution in crystals by tracing the charge transfer from initially non-interacting atomic orbitals into the chemical bonds of the final atomic configurations.

The electronic structures directly related to the chemical bonding involving the lattice, i.e., Ti and N species, were analyzed using the Crystal Orbital Hamiltonian Population (COHP). The relative magnitude of the chemical bonding is obtained based on the overlap population analysis of two chemical species used to investigate the binding character of chemical bonds in a crystal^56^. COHP was calculated by using the package LOBSTER^57,58^. Sangiovanni et al.^2^ stated a comparison and fine accuracy of VASP-PAW methods with full potential methods for a similar system and study. The basis sets used^57^ for all the chemical species in the LOBSTER analysis were presumed by the recommendation of the LOBSTER package initial run. By using this recommendation, we reduced the absolute charge spilling values below 1% for the non-magnetic systems, whereas below 3% in magnetic systems.

VESTA^59^ and pymatgen^60^ packages were used to visualize and process the structures.

## Data Availability

The datasets used and/or analyzed during the current study are available from the corresponding author upon reasonable request.
